# Radiofrequency Ablation for Treatment of Symptomatic Uterine Fibroids

**DOI:** 10.1155/2012/194839

**Published:** 2011-09-27

**Authors:** Siân Jones, Peter O'Donovan, David Toub

**Affiliations:** ^1^Department of Obstetrics and Gynaecology, Bradford Royal Infirmary, Duckworth Lane, Bradford BD9 6RJ, UK; ^2^Gynesonics, Inc., 604 Fifth Avenue, Suite D, Redwood City, CA 94063, USA; ^3^Department of Obstetrics and Gynecology, Albert Einstein Medical Center, 5501 Old York Road, Philadelphia, PA 19141, USA

## Abstract

The use of thermal energy-based systems to treat uterine fibroids has resulted in a plethora of devices that are less invasive and potentially as effective in reducing symptoms as traditional options such as myomectomy. Most thermal ablation devices involve hyperthermia (heating of tissue), which entails the conversion of an external electromagnetic or ultrasound waves into intracellular mechanical energy, generating heat. What has emerged from two decades of peer-reviewed research is the concept that hyperthermic fibroid ablation, regardless of the thermal energy source, can create large areas of necrosis within fibroids resulting in reductions in fibroid volume, associated symptoms and the need for reintervention. When a greater percentage of a fibroid's volume is ablated, symptomatic relief is more pronounced, quality of life increases, and it is more likely that such improvements will be durable. We review radiofrequency ablation (RFA), one modality of hyperthermic fibroid ablation.

## 1. Introduction

Uterine fibroids (leiomyomata uteri) are benign solid tumors that are present in the majority of women in the USA by the age of 50 [1]. While often asymptomatic, fibroids can result in abnormal uterine bleeding, pelvic pressure, pain, subfertility, dyspareunia, and other symptoms. Submucous and intramural fibroids are most associated with heavy menstrual bleeding (HMB) [2–5]; subserosal fibroids are more often innocuous unless sufficiently large so as to contribute to bulk symptoms. Many fibroids contain elements of more than one fibroid type; that is, fibroids may have submucous and subserosal components and may be transmural.

Fibroids are the most common benign female reproductive system tumor and remain the leading benign indication for hysterectomy in the USA [6, 7]. Between the years 1990 and 1997, the presence of symptomatic leiomyomata uteri was the primary diagnosis in 40.2% of all hysterectomies in the USA [6]. In the UK, fibroids are the second most common indication for hysterectomy, as approximately 30% of 42,500 annual hysterectomies are performed for fibroids [8, 9]. 

Fibroids have been reported to occur at a rate of 2.0–9.2 per 1,000 woman-years, and the incidence increases with age until menopause [7]. Women of African ancestry are at increased risk for the development of uterine fibroids, with a reported fibroid incidence of 34.4 per 1,000 woman-years in this population [7]. By the age of 50, approximately 70% of white women in the USA will have developed at least one fibroid, whereas the cumulative incidence was over 80% in one large study of black women [1].

When symptomatic, uterine fibroids are associated with a significant reduction in health-related quality of life (HRQOL) as determined by The uterine fibroid symptom and quality of life questionnaire (UFS-QOL), a validated fibroid-specific survey tool [10]. Fibroids also result in a significantly greater degree of health care utilization, including office and clinic visits. For every woman with fibroids, the average annual medical cost is $5,989 USD. This is greater than the $1,846 annual health care cost per woman without fibroids. If one includes indirect costs, such as the costs of excess absenteeism and disability claims, the total per-woman cost of fibroids amounts to $8,192 each year, which is 2.6 times the annual total health care cost for women without fibroids [11]. It has been estimated that the total annual direct cost of fibroids in the USA amounts to $2 billion [12].

Classic treatment options for symptomatic fibroids include hysterectomy and myomectomy. More recently, uterine artery embolization (UAE) has been demonstrated to be safe and effective, but the impact of this treatment modality upon fertility remains to be determined [13]. Despite the availability of suitable management choices for fibroids, there remain unmet needs. Hysterectomy does not preserve the uterus and fertility, and represents major surgery with the risk of significant complications. Uterine artery embolization is not currently recommended for women who desire future fertility, and fibroid recurrence is a possibility, with approximately 20% of patients subsequently requiring hysterectomy [14]. Myomectomy, which may be performed via laparotomy, laparoscopy, hysteroscopy, or occasionally the vaginal route preserves the uterus and fertility, but like UAE is not definitive therapy for many women. 

There has been considerable interest in the use of various forms of energy to heat and ablate uterine fibroids, including radiofrequency energy, focused ultrasound and microwaves. Unlike uterine artery embolization, which results in tissue infarction with disruption of cell membranes and spillage of intracellular contents, hyperthermic ablation results in thermal fixation, which preserves cellular architecture, as well as, coagulative necrosis [15]. 

Magnetic resonance-guided focused ultrasound (MRgFUS) utilizes focused ultrasound waves to heat and ablate fibroids, leading to fibroid shrinkage and improvement in fibroid symptoms and quality of life [13, 16–21]. However, the durability of MRgFUS beyond two years remains to be established and the availability of the procedure is currently limited. It is apparent that the clinical results of MRgFUS have greater significance and durability if higher percentages of the targeted fibroids are ablated [20].

Radiofrequency ablation (RFA) has been extensively researched as a treatment option for uterine fibroids. Medical devices utilizing radiofrequency energy are widely available and familiar to physicians. There is an established history of treating hepatocellular carcinomata and other soft tissue malignancies with radiofrequency ablation [22–31]. In the case of uterine fibroids, the presence of coagulative necrosis after treatment with RF energy can result in volume reduction of the myoma and symptomatic relief [15, 32–40]. 

It has become clear from more than two decades of clinical evidence that hyperthermic fibroid ablation, regardless of the thermal energy source, can create large areas of necrosis within fibroids that result in improved quality of life and reductions in fibroid volume, associated symptoms, and the need for reintervention. It is therefore neither necessary to perform hysterectomy nor to remove myomata in order to enhance the health and well-being of women with symptomatic fibroids. The larger the volume destroyed within a targeted fibroid, the higher the probability that treatment will be durable over the long term. While the threshold ablation volume for treatment durability remains to be established, it is apparent that when only a small portion of the fibroid is destroyed through hyperthermic ablation, the surviving fraction of the fibroid can continue to grow and symptoms can persist [20]. 

In this paper, we review the use of radiofrequency ablation in the management of uterine fibroids. All of the current RF devices have the same impact on fibroids. What differs among them are their electrode designs (bipolar versus monopolar, single tine versus multiple tines), how they are deployed (transabdominally, transcervically, transvaginally), the technique used for real-time visualization (laparoscopy, sonography), and the hardware and software that regulates energy delivery to tissue. 

Despite differences in treatment modalities, it is evident that hyperthermic energy-based systems can improve a woman's quality of life. Nonetheless, there remains an unmet need for a minimally invasive fibroid treatment that is amenable to an outpatient setting, involves a short treatment time and may be performed without the risks of general anesthesia.

## 2. Early Approaches to Hyperthermic Fibroid Ablation

The concept of ablating fibroids with hyperthermic energy, initially referred to as myoma coagulation or myolysis, was initially performed using a neodymium-doped yttrium aluminum garnet (Nd : YAG) laser to deliver energy to fibroids via laparoscopy or hysteroscopy, resulting in destruction of the local vascular supply and subsequent fibroid necrosis [41–43]. 

The development of bipolar RF needle electrodes paved the way for electrosurgical ablation of fibroids via laparoscopy. This was initially reported by Gallinat and Lueken in 1993 using their own device that was suitable for small fibroids [43]. Goldfarb developed two versions of his own bipolar needle electrode device, one of which was intended for coagulation of posterior myomata [44, 45]. 

In 1995, Goldfarb reported on his experience with RF ablation in a study of 150 women [45]. Of note, patients received neoadjuvant GnRH-analogues (GnRH-a) for at least three months to shrink fibroids at least 25% before ablation; patients who did not respond to GnRH-a treatment were offered myomectomy or hysterectomy. An average of 30–50 needle insertions were made into a fibroid, and Goldfarb reported that a 7 cm fibroid (reduced from 10 cm after the use of GnRH-a) could be treated in 20–30 minutes. It should be noted that concomitant with RF ablation, 30% of the subjects underwent a hysteroscopic endometrial ablation, 20% were treated with hysteroscopic resection of submucosal fibroids, and 37% had endometrial ablation combined with hysteroscopic myomectomy. These adjuvant procedures, along with the use of preoperative GnRH-a, confound the ability to evaluate the impact of myoma ablation on bleeding outcomes. That said, Goldfarb reported additional reductions in fibroid size at six months (as much as 50%), above what had been accomplished temporarily with GnRH-a treatment. No fibroids increased in size after treatment, and there were few complications. Two women were readmitted, one for a pelvic abscess requiring hysterectomy and the other for parenteral antibiotics due to bacteremia. Of the 150 patients, only six had pain symptoms suggestive of fibroid degeneration, and these women were managed expectantly. Three women underwent second-look laparoscopy, and all of these had mild pelvic adhesions that were managed with adhesiolysis; it was felt that there were fewer adhesions than after Nd : YAG laser ablation. One woman had been diagnosed preoperatively with a leiomyosarcoma suggested by the massive growth of a fibroid during GnRH-a treatment. It was noted that 100% of the 150 women treated with RF ablation (with or without concomitant endometrial ablation and/or hysteroscopic myomectomy) responded to these early attempts at RFA treatment of uterine myomata, with significant reductions in fibroid size at six months; all subjects were asymptomatic after treatment.

## 3. Risks and Concerns Associated with Early Methods of Hyperthermic Fibroid Ablation

Despite good reported efficacy, laser ablation and the early RF bipolar needle electrodes were not widely utilized. The transserosal use of bipolar needle electrodes and Nd : YAG laser energy was associated with serosal injury and abdominopelvic adhesions, likely due to the multiple passes through the serosa necessary to adequately treat a single fibroid with a bipolar array in the absence of real-time uterine imaging. Goldfarb suggested that, compared to the use of the Nd : YAG laser, the use of bipolar needle electrodes was associated with a decreased risk of adhesions based on small case series and one personal communication [45]. 

There have also been concerns expressed about uterine rupture during future pregnancies after these original methods of performing RF ablation of fibroids, albeit based solely on anecdote. Arcangeli and Pasquarett reported a single case of uterine rupture at 26 weeks' gestation in which the neonate subsequently died from prematurity and anemia [46]. Phillips and colleagues published their experience with 167 women who underwent either Nd : YAG laser or bipolar needle hyperthermic ablation of fibroids, with or without concomitant endomyometrial resection or hysteroscopic myomectomy; some women also received neoadjuvant GnRH agonist therapy [47]. Phillips and colleagues recommended that, because of the potential for injury to the surrounding myometrium, hyperthermic fibroid ablation should be considered only on an individualized basis in women who desire future childbearing. It is interesting to note, however, that two women in their study conceived and underwent uncomplicated, full-term vaginal deliveries. Their warning about hyperthermic fibroid ablation (i.e., the older technique of multiple transserosal punctures without concomitant imaging) is based on the case report of Arcangeli and Pasquarett, a personal communication involving two women who experienced uterine rupture at 32 weeks' gestation and at term, respectively, and case reports of uterine rupture after laparoscopic myomectomy in which electrosurgery was employed and 3–0 and 4–0 polyglactin sutures were used for uterine closure. Phillips and colleagues suggested “the use of these sutures rather than ones with stronger tensile strength such as 1 or 0 polyglactin may have been responsible for or have contributed in part to the uterine rupture.” 

Vilos and his colleagues published a report of three women who conceived against medical advice after laparoscopic RFA with bipolar needles [48]. One of the three women successfully delivered at term via Cesarean section. The two other women experienced uterine rupture at 32 weeks' and 39 weeks' gestation, respectively; the premature fetus did not survive. Vilos also took note of a report by Wood and colleagues, in which a catastrophic gravid uterine rupture at 26 weeks' gestation took place three months after bipolar RF needle ablation of a fundal fibroid; after repair, a uteroperitoneal fistula resulted [49].

Even with this very limited literature base, the possibility of uterine rupture and its attendant consequences remain a potential concern with the earlier methods of Nd : YAG laser ablation and bipolar RF needle ablation, performed without imaging guidance.

## 4. Volumetric Hyperthermic Fibroid Ablation

One of the limitations of the initial attempts at hyperthermic fibroid ablation was the inability to determine the extent of the ablation during surgery. The creation of multiple ablation sites within a given fibroid is one way to maximize the ablation volume, which in turn increases the likelihood that the fibroid will undergo sufficient volume reduction as to prevent symptoms and regrowth in the long term. However, as performed with a bipolar needle electrode system passed through the serosa, this is time consuming and adhesiogenic, due to the electrode geometry and the multiple violations of the serosal tissue. And as mentioned, repetitive, multiple unguided ablations of uterine fibroids may raise the possibility of myometrial weakening and future uterine rupture during pregnancy.

Recent RF energy delivery systems have obviated the need for multiple repetitive insertions of needle electrodes through a targeted fibroid in order to achieve an optimal ablation. Real-time sonography can provide confirmation of accurate targeting during the procedure, and the ablation volume can be tailored to an individual fibroid, minimizing the need to create more than one or two ablations in that fibroid. This is a volumetric approach to ablation, in which generally one or two ablations of predictable volume are created to destroy a desired volume of the targeted fibroid. The successful application of RF ablation to solid tumors of the liver and other organs has affirmed the validity of this approach [50, 51]. 

Image-guided, volumetric hyperthermic fibroid ablation thus obviates the use of multiple ablations within a fibroid in the absence of concurrent imaging with the potential for unintended and unrecognized ablation of surrounding myometrium. This creates a new level of safety and predictability of fibroid ablation with RF devices, and increases the probability that targeted RFA of uterine fibroids is not associated with uterine rupture and other effects on future pregnancy.

## 5. Clinical Studies of Volumetric Radiofrequency Ablation of Uterine Fibroids

The feasibility of percutaneous RF ablation under ultrasound guidance was demonstrated by Recaldini and colleagues [40]. They treated six women who had up to three symptomatic submucosal or intramural fibroids 4–6 cm in diameter using a LeVeen coaxial needle electrode. The needle electrode was placed percutaneously under ultrasound guidance. Outcome measures included the uterine fibroid symptom and quality of life questionnaire (UFS-QOL) and fibroid volume reduction as assessed by contrast-enhanced sonography. Mean followup was nine months, and median fibroid diameter and volume were significantly reduced from 4.8 cm (range, 4.4–5.2) and 58.57 cm^3^ (range, 44.58–73.58) to 2.3 cm (range, 1.2–3.2) and 8.97 cm^3^ (range, 0.90–18.81), respectively. The median symptom score on the UFS-QOL fell from 47.2 (range, 31.8–67.3) to 5.15 (range, 0–26) and the median quality of life score increased from 63.92 (range, 37.2–86.0) to 96.2 (range, 86.3–100.0). Four of the six patients were free of symptoms. 

A followup to the study of Recaldini and colleagues was published in 2009 by Carrafiello and colleagues [36]. This medium-term follow-up report involved eleven women (six from their previous report) with 1–3 symptomatic fibroids up to 8 cm in diameter. The patients underwent percutaneous, transabdominal radiofrequency ablation, again using a LeVeen needle electrode (Boston Scientific) under contrast-enhanced transabdominal sonographic guidance. Outcome measures were the UFS-QOL and fibroid volume reduction. The mean baseline symptom score was 50.30 (range 31.8–67.30), and the mean baseline quality of life score was 62 (range 37.20–86.00). The average baseline diameter of the treated fibroids was 5.5 cm (range 4.4 cm–8 cm) and their mean volume was 101.5 cm^3^ (range 44.58 cm^3^–278 cm^3^). For fibroids over six centimeters in diameter (two patients), two ablations were performed to maximize the volume of necrosis. Of the eleven women, one woman had two fibroids, only one of which was treated; the remainder had a single fibroid. After treatment, contrast-enhanced sonography indicated complete ablation of all fibroids, as evidenced by hypovascular necrosis. Mean followup was nine months (range 3–12 months), during which time the mean symptom score fell to 13.38 (range 0–67.1) and the mean quality of life score rose to 90.4 (range 43.8–100). At the last evaluation, the posttreatment mean fibroid diameter was 3.0 cm (range 1.20 cm–4.5 cm) and the mean volume was 18 cm^3^ (range 0.90 cm^3^–47.6 cm^3^). Of note, in the two subjects with fibroids over 6 cm in diameter, the reported volume reduction was 90% and this was stable at 12 months. Nine of eleven patients (81%) experienced volume reduction over 65% at six months after treatment. At the last checkup, six of eleven patients (54%) were asymptomatic, while symptoms had decreased for another four patients (36%). Thus, symptom and quality of life scores improved in 10 of 11 patients (91%). One patient, despite volumetric reduction, did not experience symptomatic improvement and underwent hysterectomy. At the time of hysterectomy, there were no adhesions noted and there were two necrotic adjacent nodules in the endometrial surface from the previous ablation. There were no complications noted and no patient required retreatment. The authors concluded that this larger cohort with longer followup confirmed previous papers from their group on the feasibility and effectiveness of this approach. The feasibility of multiple electrode deployments and ablations was also highlighted, as these were not associated with complications.

Bergamini and colleagues used a multitine RF needle electrode placed laparoscopically to ablate fibroids in 18 women with fibroids 5–8.6 cm in diameter and 14.8–332.8 cm^3^ in volume [35]. As opposed to a single needle electrode system, this multitine device was able to produce a spherical, as opposed to cylindrical, volume of ablated tissue. A single insertion was used for fibroids up to 5 cm in diameter. Outcome measures included fibroid volume reduction (as determined sonographically) and UFS-QOL score; median followup was 10 months (range 3–12). By month six, the median fibroid volume decreased by 77% (*P* < 0.01). No additional significant volume reductions were detected after that time point. Nine women were followed out to 12 months and there was no evidence of new growth. Seven of the nine subjects (77.8%) were symptom free at 12 months after treatment. At six months, median symptom scores fell from 43.7 (range 12.5–90.6) at baseline to 9.7 (range 1.1–52.8; *P* < 0.01). Median quality of life scores rose from 66.7 (range 35.0–93.9) at baseline to 100.0 (range 98.2–100; *P* < 0.01) at six months.

Ghezzi and colleagues provided data on the first 25 women treated in their center with a multitine needle electrode device [38]. All 25 women were assessed at six months, 24 through one year, 18 through two years, and 9 through three years; median followup was 24 months. Fibroid measurements were performed with sonography. Mean fibroid volumes were reduced by 65.6%, 77.9%, 78.6%, and 83.9% at 6, 12, 24, and 36 months, respectively. Mean UFS-QOL symptom severity scores went from a baseline of 43.7 to 4.7 at six months and 0 by 12 months; mean symptom scores remained at zero through 36 months of followup. Health-related quality of life (HRQOL) on the same UFS-QOL questionnaire rose from a median baseline score of 63.1 to 99.1 at six months and then to 100 at 12 months, which persisted through 36 months. There were no long-term complications reported. One woman underwent hysterectomy for recurrent fibroid symptoms at 12 months after treatment despite a significant reduction in fibroid volume. 

Cho et al. have employed a single, straight 25 cm 18-gauge radiofrequency ablation needle electrode to manage symptomatic uterine fibroids [37]. The needle electrode was generally placed transcervically; the posterior cul-de-sac or anterior vesicouterine fold was also utilized in selected subjects. Targeting of selected fibroids was carried out using either transabdominal or transrectal sonography. Experience with this system has been reported for 153 women, 14 of who were excluded due to lack of followup. Mean pretreatment dominant fibroid volume, as determined by sonography, was 65.12 cm^3^; multiple ablations were performed to treat fibroids larger than 5 cm in diameter. By 18 months, the mean dominant fibroid volume was 19.3 cm^3^ (73% reduction). The vast majority of the reduction in dominant fibroid volume occurred within the first 12 months. For fibroids with a pretreatment volume of 75 cm^3^ or less, mean UFS-QOL symptom severity scores went from 46.9 to 4.2 at 18 months (*P* < 0.05). Mean health-related quality of life scores rose from 66.9 to 97.7 at 18 months (*P* < 0.05). Six women (4.3%) underwent reintervention during this period, and women with fibroids with a volume >75 cc (5.3 cm in diameter) were more likely to require reintervention and have lower satisfaction scores. It should be noted that of the six women who were retreated, five had reintervention prior to the 12-month followup and the single reintervention at 16 months was due to a *de novo *fibroid that was asymptomatic but had a more rapid growth rate. Long-term complications by 18 months were also uncommon and minor, consisting of spotting up to 8 weeks after ablation in 9.6% of women. There were no major complications, such as injury to the bowel or bladder. 

Szydłowska and Starczewski published their experience with performing radiofrequency ablation of uterine fibroids in 46 women with a monopolar needle electrode delivered laparoscopically [34]. Their procedure involved making a single puncture for fibroids with diameters ≤3 cm and two punctures through the serosa for fibroids >3 cm in diameter, with three 10-second ablations per puncture. Followup at six months, which included Doppler velocimetry, produced striking results. In 73.3% of women who had fibroids with volumes less than 5 cm^3^(roughly corresponding to diameters of 2 cm and less), their fibroids were undetectable on sonographic imaging at six months. In the remaining 26.7% of women with initial fibroid volumes of 5 cm^3^ or less, their fibroids were reduced in mean volume by 83.5% (*P* < 0.001). For women with initial fibroid volumes over 5 cm^3^, 41.9% of subjects had undetectable fibroids at six months, with 75% mean shrinkage noted in the remaining cohort (*P* < 0.001). Fibroid volume reduction correlated with increases in the pulsatility index (PI) and resistance index (RI) in both uterine arteries at six months, signifying a reduction in blood flow to treated fibroids. There was symptomatic relief of HMB and/or pelvic pain in 88% of patients by six months. One subject was refractory to the effects of fibroid ablation, with no diminution in size of her fibroid and the appearance of two new fibroids; she was managed with open myomectomy, and no adhesions were noted at the time of laparotomy.

Recently, Kim and colleagues have reported results of RF ablation in 69 women with fibroids up to 12.5 cm in diameter, some of who desired fertility [39]. They used a single RF needle electrode that was saline cooled, to prevent carbonization, and inserted it transvaginally under conscious sedation with transvaginal ultrasound guidance. In the 10.1% of women who desired future fertility, the needle electrode was purposely placed so as to avoid the endometrium. Outcomes were assessed at 1, 3, 6, and 12 months after ablation. Fibroid volumes were measured by transvaginal sonography, and HMB was evaluated by a tabulation of the number of soaked normal-sized sanitary products in a menstrual cycle; overall symptoms were assessed with the UFS-QOL symptom severity score (SSS) questionnaire. There were significant improvements in heavy menstrual bleeding noted at each assessment (1, 3, 6, and 12 months; all *P* < 0.001 versus baseline). Overall symptoms, as measured with the UFS-QOL SSS, were also significantly reduced at all assessments. Finally, there were three reported uncomplicated pregnancies; two normal spontaneous vaginal deliveries and one Cesarean section. This paper by Kim and colleagues is particularly important as it represents the first bleeding study of any hyperthermic ablation technique, and also provides pregnancy data (albeit with a very small population). Given the small number of patients who became pregnant, it remains unproven if current RF ablation methods would avoid the occasional cases of uterine rupture reported after the earlier work with both Nd : YAG laser and bipolar needle myolysis. That said, the early evidence is promising. Unlike the earlier methods of myoma coagulation that involved multiple ablations of fibroids without imaging guidance, today's volumetric, image-guided ablation permits the operator to ablate a fibroid with a single ablation in most cases, and often in a fashion that confines the ablation to the myoma and spares the surrounding myometrium and endometrium. For additional support, there have been more than 50 reported cases of pregnancy after MRI-guided focused ultrasound, another form of hyperthermic ablation, with generally good outcomes and no reports of uterine rupture [52–55].

Finally, intrauterine ultrasound-guided radiofrequency ablation of fibroids is a procedure that has been reported to be safe and reliable in a cohort of 19 women [56]. This technique involves the use of the VizAblate System, which combines RF ablation for treatment with intrauterine sonography for imaging in a single device that is inserted transcervically. There were no complications when the device was used to ablate 20 fibroids in the 19 subjects. After either immediate or delayed (16-17 days after ablation) total abdominal hysterectomy, the extirpated uteri were stained with triphenyltetrazolium chloride (a vital stain to discriminate between viable and nonviable tissue). This vital staining indicated that in these patients, 67.2% ± 27.0% of the fibroid volume was successfully ablated (range 15–100%; median 75%). As noted previously, it is not necessary to completely ablate fibroids in order to provide symptomatic relief. An efficacy study involving the VizAblate System is currently in progress in Europe and Mexico.

There has been no reported experience with preoperative use of GnRH agonists in the setting of RF ablation, outside of the early experience with what had been termed “myolysis.” Some recent studies of RF ablation in uterine fibroids specifically excluded women who had received GnRH agonist therapy [37, 38]. That said, there is no obvious reason why pretreatment with GnRH agonists would not be helpful when considering large fibroids for treatment with RF ablation. Large fibroids may pose a challenge for both ablative and embolizational therapies. It is unclear what precisely qualifies a fibroid as “large.” Regardless, it is thought that increased tissue impedence and vascularity may limit the ability of hyperthermic ablation therapies to destroy large fibroids, even when multiple, overlapping ablations are created in a single fibroid [37]. Thus, preoperative use of GnRH agonists may enable the wider use of RF ablation with large fibroids.

Reintervention rates, which are perhaps more clinically meaningful than fibroid volume reduction, have been reported to be under 10% in several studies from 3–36 months. [36–38] While additional study is necessary to clarify the recurrence and reintervention rates after RF ablation of uterine fibroids, the initial evidence base is favorable.

## 6. Conclusion

The use of radiofrequency energy has a demonstrable ability to successfully and safely ablate a range of fibroid volumes. Earlier management with RF ablation has been limited by the lack of concurrent imaging and the need for multiple fibroid punctures resulting in serosal injury, adhesions, and potential myometrial disruption during pregnancy. More recent volumetric techniques, in concert with sonography, minimize the need for multiple punctures through fibroids; in the case of transcervical or transvaginal RF ablation, the serosa is entirely avoided. 

Hyperthermic fibroid ablation results in thermal fixation and coagulative necrosis within the fibroid. When a sufficient percentage of a fibroid has been ablated, a reduction in fibroid volume and associated symptoms can be realized. Such symptomatic relief appears to be durable, but additional, longer studies are required to more fully ascertain the reintervention rate after RF ablation of uterine fibroids. The emerging clinical literature base indicates that patients will reliably experience significant reductions in fibroid volumes and symptoms as a result of radiofrequency ablation.
